# Global diagnosis of land–atmosphere coupling based on water isotopes

**DOI:** 10.1038/s41598-023-48694-1

**Published:** 2023-12-03

**Authors:** Ruiqiang Yuan, Fei Li, Ruyu Ye

**Affiliations:** 1 School of Environment and Resource Sciences, Shanxi University, Taiyuan, China; 2 Institute of Geographic Sciences and Natural Resources Research, Chinese Academy of Sciences, Beijing, China

**Keywords:** Environmental sciences, Hydrology

## Abstract

Land–atmosphere coupling (LAC) plays a significant role in weather and climate and is related to droughts and heatwaves. We propose a simple and efficient LAC diagnosis method based on the analysis of water isotopes in atmospheric water vapour and precipitation. Using the method, we identify the primary LAC hotspot regions of the globe and reveal the seasonality of LAC strength. We find that LAC strength exhibits a relationship with latitude. Low latitudes present stronger LAC strength and contribute more significantly to the overall LAC area compared to boreal middle and high latitudes. It's important to note that LAC primarily manifests in the troposphere and is detected in the lower stratosphere of low latitudes, with limited influence observed in the stratosphere. However, the impact of LAC is noticeable in the upper stratosphere in boreal middle and high latitudes. Moreover, the seasonality of LAC strength is pronounced. On a global scale, the season with the strongest LAC is boreal autumn in the Northern Hemisphere but boreal summer in the Southern Hemisphere. Notably, this pattern does not exhibit a seesaw effect between the two hemispheres. Our isotope-based LAC diagnosis method captures the major LAC hotspots found in previous work and validates the seasonality of LAC within these hotspots. This substantiates the reliability and effectiveness of our isotope-based approach.

## Introduction

The land–atmosphere coupling (LAC) investigates the interactions between the subsurface, land surface, and the atmosphere through the exchange of mass and energy, affecting several key mechanisms including boundary layer mixing, convection, cloudiness, or precipitation^1,2^. Cioni and Hohenegger noted that precipitation is expected to increase with enhanced evaporation regardless of atmospheric state^3^. LAC can amplify heat extremes under declining soil moisture^4^. The soil state modulates LAC duration^5^. The wet-dry transition zone often has the strongest LAC^6–9^. The impact of LAC extends to the occurrence of extreme weather events, such as heavy rainfall, flooding, and droughts. It has been projected the frequency and intensity of concurrent soil drought and atmospheric aridity will greatly increase in the twenty-first century^10^. A comprehensive understanding of LAC is crucial for assessing and mitigating the risks associated with such events.

However, despite its critical role in a changing world, measuring LAC directly in the real world has proven to be a formidable challenge^11^. Previous studies have relied on physical and statistical models to explore LAC, with various diagnostic methods developed. For instance, the CTP-HI (the Convective Triggering Potential and Humidity Index) framework can identify and classify LAC^12^. However, the framework relies on intensive and costly observation. The ability of physical models to capture the coupling signal relies on a reasonable representation of near-surface conditions^13,14^ and a relatively high resolution^5^, which limits the application of the physical model. The correlation between two characteristic variables related to the surface exchange process can be considered a useful LAC metric^15^. Thus, statistical models can be used to diagnose LAC. Soil moisture (SM) is one of the most important variables of the climate system as it constrains evapotranspiration, affecting the surface energy and water balance^16^, and is therefore widely employed for the identification of LAC^17^. The relationship between SM and surface temperature^18^, between SM and surface fluxes^1^, between SM and evapotranspiration^19^, and between SM and evaporative fractions (EF)^20^, and so on allows us to identify LAC. However, the inherent difficulty and relatively low accuracy of SM measurements have restricted the global application of these SM-based statistical models. Recently, multiple environmental factors were used to achieve a more accurate diagnosis of LAC^9,20^, which generally requires a large amount of data. As it stands, LAC diagnosis remains a significant challenge.

LAC plays a pivotal role in the evolution of weather and climate^21^, water-related disasters, and multi-feedback mechanisms within the Earth's complex system. Nevertheless, the lack of high-quality, long-term, and globally distributed observations has impeded robust and realistic global LAC identification^22^. In response to this challenge, our study aims to introduce a novel, straightforward, precise, and efficient method for diagnosing LAC on a global scale. Importantly, our method distinguishes itself from existing approaches as it is the first isotope-based LAC diagnostic method. The results we present hold the potential to advance LAC diagnosis and deepen our understanding of this critical Earth system process.

## Methodology

### Theory

Isotope fractionation is defined as the phenomenon that the isotope ratio of an element in a certain compound changes by the transition of the compound from one physical state or chemical composition to another. The progressive rain out of the vapour masses is a main process in the global water cycle, concerning the water isotopes fractionation. Once the vapour has been formed, the rainout process proceeds in isotopic equilibrium, when the isotope fractionation between liquid water and vapour is written as:

1 $${\text{H}}_{2} {\text{O}}_{{\text{L}}} \; + \;{\text{H}}_{2} {\text{O}}*_{{\text{V}}} \leftarrow \to {\text{H}}_{2} {\text{O}}*_{{\text{L}}} \; + \;{\text{H}}_{2} {\text{O}}_{{\text{V}}}$$

where * stands for the rare isotopic species containing ^2^H(D) or ^18^O. The equilibrium isotope fractionation factor *α* is defined by the equilibrium constant of this exchange reaction^23^. Specific values for the fractionation factors of oxygen and hydrogen isotopes are given as a function of the temperature^24^. Therefore, the water isotope composition in vapour (*R*_V_) is highly correlated with the composition in raindrops (*R*_L_).

The reservoir of atmospheric moisture in an air column is very dynamic, quickly responding to changes in external conditions. First, the mean turnover time (calculated by dividing the number of materials present in a system by the into or out flux rate for the materials) of water vapour in the global atmosphere is around 10 days concerning the net evaporation or precipitation fluxes. Second, the sources of water vapour are various, including oceanic evaporation vapour, terrestrial evapotranspiration vapour along the moisture trajectory, and locally recycled vapour^25,26^. Both the oceanic and the terrestrial vapour belong to the external water cycle. Locally recycled vapour belongs to the internal water cycle. Besides, rainfall evaporation produces a minor part of atmospheric moisture^27^. The moisture originating from the external water cycle can be understood in terms of large-scale mixing among convective air parcels that have undergone various condensation and moistening processes^23^. Oceanic and terrestrial vapors in the external water cycle exhibit high dynamism, partly due to the short turnover time of atmospheric moisture. In addition, only a part of the moisture in an air column leads to precipitation over an extended period. For these reasons, the isotopic correlation between atmospheric vapour (*R*_V_) and precipitation (*R*_L_) is weak on a monthly scale, despite often being robust during storm events.

However, LAC can enhance the correlation between *R*_V_ and *R*_L_ on a monthly scale. Soil moisture is usually recharged by precipitation. Signals of precipitation can be retained in soil moisture for days or months, a phenomenon known as soil moisture memory. Globally, the average soil moisture memory extends to about one month^11^. This implies that the isotopic signal of monthly precipitation can persist due to soil moisture memory. During the LAC, the locally cycled moisture that originated as evapotranspiration from soil moisture most recently from a specified area forms a part of precipitation falling over the same area^11^. The LAC enhances the local vapour cycling. However, because locally recycled vapor differs isotopically from the ambient water vapor in the external water cycle within an air column^28^, an increase in locally recycled moisture in an air column can strengthen the isotopic correlation between atmospheric vapor and precipitation on a monthly scale. Consequently, it is expected that strong LAC will promote a high correlation between monthly atmospheric vapor and monthly precipitation. For the same reason, LAC can be identified through the isotopic correlation between monthly precipitation and atmospheric vapor.

### Data and method

Global monthly vertical profiles of HDO in the atmosphere were measured by the Tropospheric Emission Spectrometer (TES) aboard the Aura spacecraft. The TES data set is currently the most abundant water vapour isotopologue data set, which was provided at 2-degree latitude by 4-degree longitude spatial grids and at a subset of pressure levels (825, 681, 464, 316, 215, 147, 100, 68, 46, 32, 22, 15, 10, 7, and 5, unit: hPa). The Level 3 TES data are mostly sensitive to the 550 to 800 hPa layer, which is the area where most atmospheric water vapour is concentrated. Therefore, we employed the TES monthly HDO products of 825 hPa and 621 hPa to diagnose LAC.

SWING2 (the Stable Water Isotope Intercomparison Group, Phase 2) is a project to compare water-isotope-enabled general circulation model results across modeling groups. It was reported that simulations of HDO in precipitation produced by isoGSM (the Isotopes-incorporated Global Spectral Model) show lower standard deviation, RMSE (Root Mean Square Error), and higher correlation with GNIP (the Global Network of Isotopes in Precipitation) observations of water isotopes in precipitation^29,30^. Thus, we downloaded monthly simulations (2006–2009) of global precipitation isotopes produced by isoGSM (nudged).

The TES data were resampled to 1.875° × 1.889° (longitude × latitude) following isoGSM’s spatial resolution. To do a global diagnosis of LAC hotspots, Pearson’s *r* between TES monthly water vapour HDO at the pressure levels 825 hPa and 621 hPa and isoGSM monthly precipitation HDO were calculated at every grid point, respectively, and then the larger r (*p* < 0.05) was kept as the final result. The sample size for the correlation analysis was 48. Pearson’*r* > 0.5 (moderate correlation) was considered as the indicator of the occurrence of obvious LAC. Furthermore, strong LAC regions were recognized by *r* > 0.7 (strong correlation)^31^. Similarly, LAC was detected using Pearson’s r for every season. The sample size for each season was 12. The seasonality of LAC was recognized by the seasonal total areas of LAC regions and strong LAC regions. In addition, to test the effect of LAC on the troposphere and stratosphere, Pearson’s *r* between monthly water vapour HDO at all pressure levels and monthly precipitation HDO were calculated according to the above procedure. Data processing and analysis were executed using NCL (NCAR Command Language).

We also downloaded data on precipitation, humidity, and air temperature (CRU TS V4.03, 0.5° × 0.5°, monthly) from the Climatic Research Unit and 0–40 cm soil moisture content data (GLDAS NOAH10 V2.1, 1° × 1°, monthly). Based on these data, we derived the 30-year average of monthly precipitation, humidity, air temperature and the 20-year average of monthly soil moisture content for recognized LAC hotspots.

## Results

### Hot spots of land–atmosphere coupling

**Figure 1:**
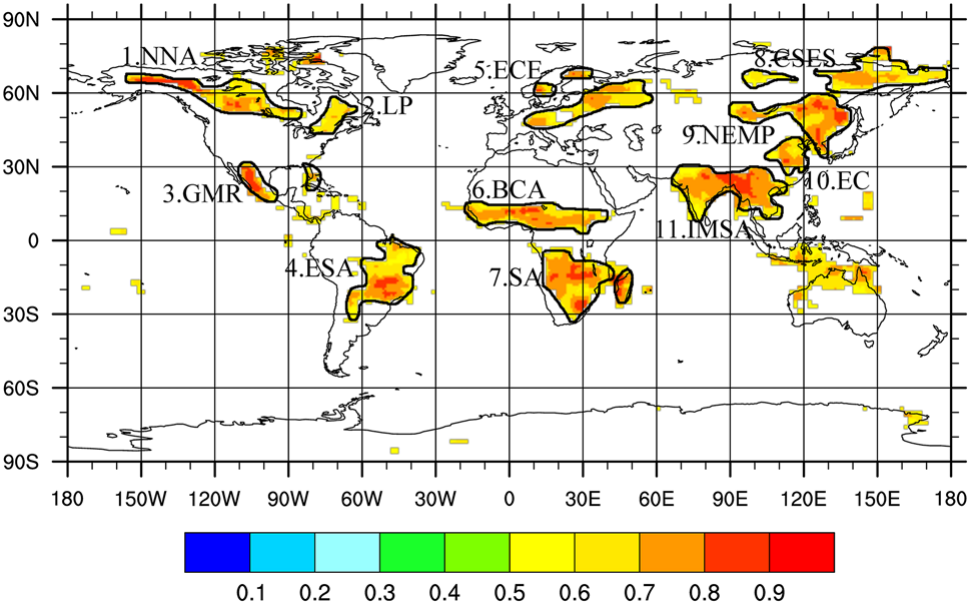
Hot spots of land–atmosphere coupling (LAC) indicated by the HDO correlation between precipitation and vapour. The map was generated using NCAR Command Language (NCL Version 6.4.0). See http://www.ncl.ucar.edu/ for more details.

Eleven LAC hotspot regions were recognized (Fig. 1 & Table S1), including (1) the northern part of North America (NNA), (2) the Labrador Peninsula (LP), (3) the Gulf of Mexico rim (GMR), (4) the eastern South America (ESA) including Cerrado, Caartinga and Chaco, (5) the eastern and central Europe (ECE), (6) the belt region in central Africa (BCA), the Sahel, (7) the southern Africa (SA), (8) the central Siberian plateau and the East Siberian highlands (CSES), (9) the north and east of Mongolian plateau (NEMP), (10) the eastern China (EC), and (11) the India and the Mainland Southeast Asia (IMSA). The total area of LAC hotspot regions, 5.23 × 10^7^ km^2^, accounts for 35% of the global terrestrial area. The area of strong LAC regions accounts for 44% of the LAC hotspot regions.

The LAC hotspot regions cover various physical geographic zones, except for tropical rain forests, aridity zones, and terrestrial glaciers and ice sheets. Annual average precipitation and air temperature of the regions vary extensively from 295 to 1499 mm/yr and from 2.5 to 26.5 °C, respectively. The annual average humidity changes in the same direction as the annual average air temperature (Fig. S1). The LAC hotspot regions can be divided into two groups according to the climate condition differences. The first group includes GMR, ESA, BCA, SA, and MSA, which are located at low latitudes (30°S**–**30°N) and characterized by plenty of energy input and available water. The seasonal variation of precipitation is significant, while the seasonal variation of air temperature is insignificant. The second group includes the rest six regions, which are located at boreal middle and high latitudes and characterized by moderate precipitation and low annual average air temperature (Fig. S1). Most of the precipitation and high temperatures occur in the boreal summer for the second group.

In terms of area, 63.5% of LAC hotspot regions and 69.1% of strong LAC regions are contributed by the low-latitude group. The strongest three LAC regions belong to the low-latitude group including IMSA, SA, and BCA. IMSA has the biggest area and the highest proportion of strong LAC regions (64%). The weakest three LAC regions belong to the boreal middle-high-latitude group including LP, CSES, and EC. LP has the smallest area and the lowest proportion (15%). Therefore, low latitudes are considered the main zone of LAC.

Many LAC hotspots worldwide are influenced by monsoons. The North America Monsoon affects GMR, the South America Monsoon controls ESA, the West Africa Monsoon dominates BCA, the South Africa Monsoon prevails in SA, and the Asian Monsoon influences IMSA, EC, and NEMP. Only four high-latitude LAC hotspots remain unaffected by monsoons.

### Seasonality of land–atmosphere coupling

**Figure 2:**
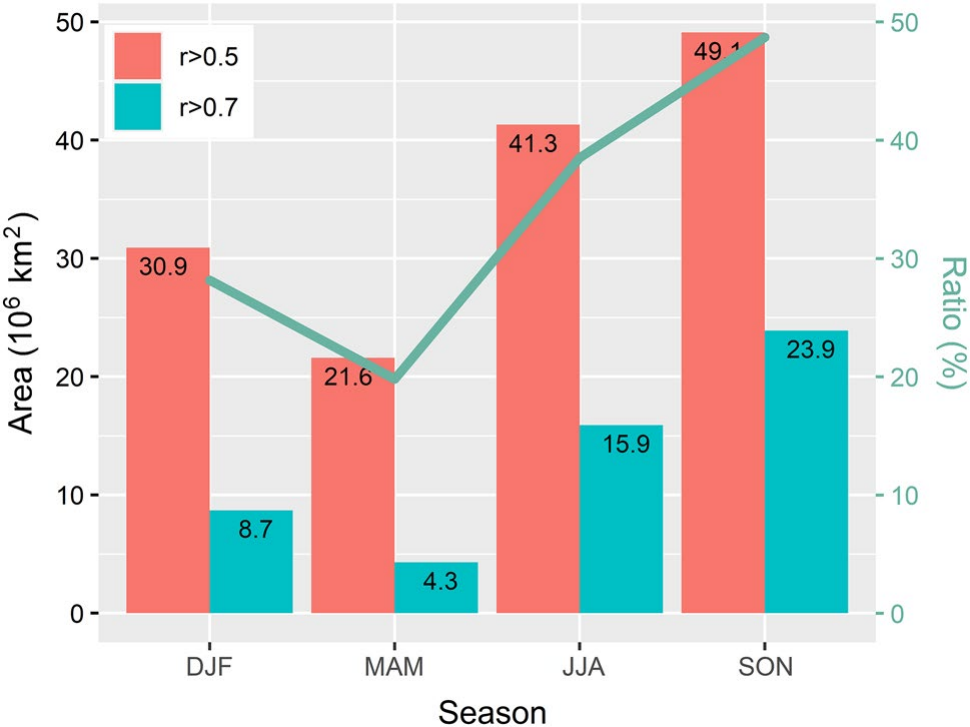
Global areas of LAC region (r > 0.5) and strong LAC region (r > 0.7) and the area ratios of strong LAC regions over LAC regions.

The seasonality of LAC strength is significant. Globally, boreal autumn and summer are the periods with strong LAC strength (Fig. S2). The strongest LAC strength occurs in boreal autumn, when the areas of LAC and strong LAC regions are 49.1 × 10^6^ km^2^ and 23.9 × 10^6^ km^2^, respectively, reaching the annual peaks (Fig. 2). The season with the weakest LAC strength is boreal spring. The area of strong LAC regions in autumn is 5.60 times higher than in spring, suggesting the remarkable seasonal variation in LAC strength. Additionally, the area percentage of strong LAC regions in LAC regions increases from 19.8% in spring to 48.7% in autumn (Fig. 2), which is also considered a result of the LAC seasonality.

The hotspot regions, NNA, GMR, BCA, CSES, NEMP, EC, and IMSA, show the strongest LAC strength in autumn, which are all located in the northern hemisphere. Among them, CESE and IMSA’s summer as well as NNA and EC’s winter exhibit strong LAC strength. In the seven regions, summer precipitation accounts for about half of the annual precipitation, while autumn precipitation is moderate, suggesting that excessive precipitation may hinder the further enhancement of LAC strength. The hotspots with strong LAC strength appear in regions with intermediate climatological soil wetness^7^. Excessive precipitation can result in excessive soil moisture, which may even turn a LAC region from a humidity- to an energy-limited region. Therefore, excessive precipitation can both temporarily and, in some cases, more persistently weaken the strength of LAC, particularly if it leads to soil saturation, flooding, or changes in vegetation cover. However, excessive precipitation can have complex effects on the strength of LAC. The specific impact can vary depending on. The LAC strength of ESA, ECE, and SA is the strongest in boreal summer. ESA and SA are located in low latitudes of the southern hemisphere, where energy input is plenty with less precipitation in the boreal summer. ECE is located at middle-high latitudes with obviously high air temperatures in summer. The LAC strength depends on the seasonal evolution of climatic conditions^19,32^. However, moderate precipitation and enough energy input are necessary for strong LAC.

## Discussion

Our method successfully identified almost all primary LAC hotspots on a global scale. Our method identifies most of the soil moisture and precipitation (SM-P) coupling regions found by Koster et al.^33^. Based on the CTP-HI framework, Alaska, central Brazil, Eastern Europe, and Russia were recognized as strong coupling regions by Ferguson and Wood^34^, which is consistent with our result. Besides, our method identified the Indochina region, north-northeastern China, and Siberia–northern Mongolia region as the LAC hot spots where the strong soil moisture-temperature (SM-T) or soil moisture–evapotranspiration (SM-ET) coupling was demonstrated^19,35,36^. Generally, the LAC hot spots revealed by our method cover almost all regions with strong SM-P, SM-T, SM-ET, or CTP-HI couplings obtained by various methods^1,7,37–41^. A large range of soil moisture variation is a necessary condition for a strong LAC^35^. Furthermore, there are different correlations between LAC coupling strength and soil moisture anomalies over areas with a normally dry or wet climate^7^. In humidity-limit regions, strong ET will induce an obvious decline of SM, showing the SM-ET coupling. In energy-limit regions, strong ET will induce an obvious decline of adjacent air temperature, showing the SM-T coupling. Increased SM could also promote precipitation by an enlarged ET flux, showing the SM-P coupling in transition zones between wet and dry regions or transition periods between wet and dry seasons^37^. No matter what type the LAC is, the strong ET is the bond between land and atmosphere in the LAC processes. The evaporated SM brings the isotopic signal of water that usually was stored in the soil at monthly scales into atmospheric moisture and subsequently the precipitation. The SM-P, SM-ET, and SM-T couplings can therefore be captured based on the correlation between isotopic compositions of water vapour and precipitation.

Our method reveals similar LAC seasonality with previous studies in those hotspots. In central Europe, the LAC in Summer is strong, which further strengthens heatwaves^42^. Afforestation and deforestation modified the LAC in Eastern European Plain and parts of Scandinavia and Eastern Europe by changing the surface flux partitioning^43^. Specially, increased LAC of SM-P was documented in areas of continuous Eurasian permafrost^44^. LAC in the other seasons is much weaker than in summer^13^, which is consistent with our result. The Indo-Gangetic Plain is one of the most densely populated areas on Earth, where summer is the prime LAC season^45^. Indochina region in spring and summer, and the Indian subcontinent in summer and fall were identified as LAC hot spots based on the soil moisture’s impact on temperature, evaporation, and EF^35^. According to our result, the Indochina region in summer and autumn, and the Indian subcontinent in autumn present significant LAC. The lower-middle reaches of the Yangtze River, North China, and Northeast China present strong LAC in summer and fall^9,19,46^. According to our result, significant LAC still occurs in the Huaihe River Catchment and the lower-middle reaches of the Yangtze River in winter. Temperature-related couplings are stronger in winter in humid areas^8^, which might be a possible explanation.

In North America, GMR includes southeastern Arizona–New Mexico in the U.S. and Northwest Mexico, the transitional zones between dry and wet climates, and U.S. Southeast, the eastern North American monsoon region. LAC in the U.S. Southeast is strong in summer, which is important for drought recovery^47^. In autumn when the monsoon nearly finishes, the areas of strong coupling primarily dominate the majority of the western part of northwestern Mexico where surface fluxes are suitably high but still sensitive to soil moisture^48^. Our result is similar to the previous results. The Great Plain of North America was recognized as a strong SM-P coupling region^33^. Recent observations show moderate to weak LAC in the Southern Great Plains in summer^49–51^. However, there is no LAC signal captured by our method, suggesting no LAC at least during our observation period. Semi-arid and arid continental climates dominate in the Great Plains. Droughts further threaten water availability in the region^52–54^. During our observation period, mega-droughts happened in the Southern United States, which could induce soil moisture depletion. Subsequently, the atmosphere is predisposed to limit precipitation and therefore not conducive to LAC.

In South America, recent work demonstrated that Amazon, Cerrado, Caatinga, and La Plata showed the strongest coupling in boreal winter, the wettest months^55^. Our result confirms that Cerrado, Caatinga, and Chaco are the LAC hotspots in boreal summer and autumn, the dry season. The spatial extent of the LAC region, based on our method, closely aligns with previous results in South America. The only exception is the eastern part of La Plata, which is not identified as an LAC region in our findings. Nevertheless, there are differences in the seasonality of LAC strength between Baker's results and ours, even though strong LAC is also observed in the boreal autumn (SON) in Baker's research. Cerrado, Caatinga, and Chaco are seasonally dry subtropical regions of woody savanna and serve as transition zones between wet and dry climates^55^. Moisture transport from lower latitudes sustains intense convective storms^56^. LAC usually tends to be significant over wet-dry transition zones^57^. The climate in these wet-dry transition zones is sensitive and subject to frequent changes. The seasonality of LAC strength can also vary from year to year due to climatic fluctuations. The difference in study periods partially accounts for the differences observed in the seasonality of LAC strength when comparing our results to previous findings. This underscores the importance of considering the impact of climate variability when interpreting these differences.

In the Western Sahel of Africa, LAC occurs in spring, just before the beginning of monsoon season^57^. For the whole Sahel (the belt region in central Africa, BCA), a strong LAC occurs in summer^1,58^. However, in our findings, the area covered by LAC regions is 1.7 times larger in autumn compared to summer, with an even more pronounced 2.7-fold increase in the area of strong LAC regions. Our isotope-based LAC diagnosis method identifies autumn as the season with the strongest LAC strength. On average, summer rainfall in BCA amounts to approximately 444 mm brought by the West African Monsoon, while autumn experiences an average of 257 mm (Table S2). The summer easterly moisture flux brings in ample water vapor and precipitation for the region, leading to an increase in average soil moisture content from 81.8 in spring to 103.0 kg/m^2^ in summer (Table S3). However, as previously mentioned, excessive summer precipitation can lead to an abundance of soil moisture, potentially transforming an LAC region from humidity-limited to energy-limited. Additionally, excessive summer rainfall suppresses evapotranspiration due to the moist atmosphere. Consequently, the LAC strength during summer with excessive precipitation is weaker than that in autumn with more moderate rainfall. Müller et al.^5^ have reported that soil memory contributes to maintaining the significant SM-P coupling in autumn, particularly when the easterly moisture flux in the Sahel is weaker. This finding supports our results. The high correlations observed between soil moisture (SM) and other characteristic variables, such as precipitation (P), may be attributed to processes other than LAC during summer. Consequently, methods based on the correlations with SM tend to overestimate LAC strength in the summer months. Further studies are needed to develop deeper into this aspect.

For the southern African hotspot, SA, remarkable LAC occurs except for boreal spring, and reaches the most extent in summer, which is in agreement with the result of Müller et al.^5^. However, some studies noted that LAC is strongest in boreal winter^1,37,58^. Lorenz et al.^41^ also indicated that strong coupling is mainly constrained to the Southern Hemisphere in boreal winter, excluding rain forests. Although LAC also occurs in the Southern Hemisphere in boreal winter according to our results, boreal summer and autumn are still the main LAC periods. We believe that a see-saw pattern of LAC seasonality between the two hemispheres is unnecessary. In the Southern Hemisphere, oceans are dominated. Lands mainly distribute in low latitudes under tropical and subtropical climates without a clear four-season division. Moreover, conditions of water and energy in low latitudes are similar.

There are still disagreements between our results and previous studies. The disagreements may be related to different spatiotemporal scales and environmental changes. The land surface to the atmosphere feedback is more significant as time scales increase from daily to monthly, indicating more significant coupling at longer time scales^59^. Similarly, from yearly to monthly, weekly, and daily scale, the correlations between SM and VPD are generally decreasing^60^. Instantaneous perturbation of precipitation would have a larger impact on LAC at a shorter time scale^59^. At the same time, high-resolution GCMs would improve the simulation of LAC due to the primary role of atmospheric conditions in LAC^5^. The differences in land use, anthropogenic activities, rooting depth, and soil type impose significant influences on LAC^49^, which could be reflected on a finer scale. The composition of temporal and spatial resolutions varies among studies, which is possibly responsible for the disagreement among LAC results. On the other hand, China has experienced substantial changes in vegetation cover, with increasing cropland in North China and forest in South China, which is considered to impose an influence on the seasonality of LAC^46,61^. In Southeast Asia, the LAC strength increased due to deforestation^62^. Cropland/grassland depletes soil moisture more readily than forests, thereby triggering a more rapid release of sensible fluxes^42^. Afforestation and deforestation modified the atmospheric humidity and stability by changing the surface flux partitioning^43^. Besides, in a warming world, an increase in boundary layer moisture in response to increased latent heat fluxes over areas of continuous Eurasian permafrost increases precipitation and low-level cloudiness^44^. Aerosols are also found to influence LAC over north-west India by modulating net radiation^45^. In the changing world, LAC is projected to increase across most of the globe^63^. A rapidly changing environment introduces interannual variation of LAC, which is responsible for the disagreement.

**Figure 3:**
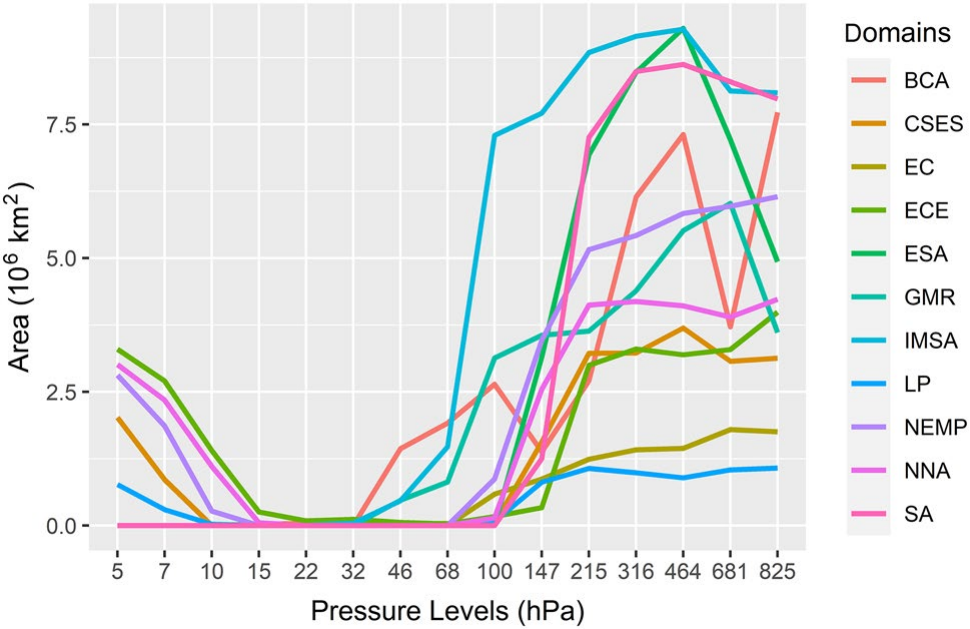
Area of LAC domains at pressure levels.

ET-LCL (lifting condensation level) coupling is a key process that determines the soil moisture-precipitation coupling^59^, which suggests there is an upper limit in the atmospheric segment of LAC. The effects of changing soil conditions on atmospheric moisture can be evident at 500 hPa^44^, which corresponds to the middle troposphere. Previous results were limited by observations. The results might have been underestimated. We derived LAC area at different pressure levels for every hotspot domain (Fig. 3). Results show that LAC in the hotspot domains was evident below 100 hPa, namely, the whole troposphere. Strong ET and intense atmospheric convection jointly spread the isotopic signal that originally is stored in soil moisture at monthly scales to the whole troposphere. For the low-latitude hotspot domains, including BCA, GMR, and IMSA, the LAC signal can even be detected as far aloft as 50 hPa in the lower stratosphere, which is related to atmospheric deep convection and high altitude of the tropopause in low-latitude region. In NNA, LP, ECE, CSES, and NEMP, the LAC signals are restored to a certain degree at pressure levels of 5–7 hPa, which is unexpected. It should be noticed that the five hot spots are all mid-high-latitude regions. On the one hand, only polar stratospheric clouds (made of ice) appear in the lower stratosphere (15–25 km) in the high latitudes. On the other hand, the stratospheric humidity has doubled over the past half century^64^, although the stratosphere contains little water vapour. It is, therefore, a reasonable inference that there is sufficient water vapour (for the observation of TES) in the upper stratosphere of the mid-high-latitude region due to higher temperature (maximum 3–17 °C) compared to the lower stratosphere. However, we must point out that the detected LAC-like signal in the upper stratosphere of mid-high-latitude hot spots does not validate the LAC in the stratosphere. Adequate water vapor transport from the troposphere to the stratosphere, diffusion of water vapor within the stratosphere, and a prolonged residence time of water vapor at these altitudes are all likely contributing factors to the emergence of these LAC-like signals. Stratospheric water remains an open issue. More observations and modeling are needed to resolve this issue.

The newly proposed isotope-based LAC diagnosis method relies on water isotope data in both precipitation and atmospheric water vapor. A long-term series of the isotope data is essential for a precise diagnosis because a sample size larger than 30 is optimal for the correlation analyses used in the method. However, for the large-scale study, TES and SWING2 serve as indispensable data sources. TES provides data from 2004 to 2018. At the same time, SWING2 offers data from 1979 to 2009. To maintain data continuity and integrity, we opted to utilize data from 2006 to 2009 for both sources. This results in a sample size of 12 for seasonal LAC diagnosis, introducing a source of uncertainty in the seasonal LAC outcome. Nonetheless, our study produces comparable and reasonable results, highlighting the potential of an isotope-based LAC diagnosis method despite data limitations. The introduction of our innovative LAC diagnostic method into the scientific community is poised to spark greater interest and stimulate further research efforts.

## Conclusions

In this paper, we proposed a novel LAC diagnosis method based on water isotopes in vapour and precipitation and verified the method at a global scale for the period from 2006 to 2009. Our method successfully identifies LAC hotspot regions and the seasonality of LAC strength. The total area of the LAC hotspot regions, 5.23 × 10^7^ km^2^, accounts for 35% of the global terrestrial area. The area of the strong LAC regions accounts for 44% of the LAC hotspot regions. There are no LAC in tropical rainforests, aridity zones, terrestrial glaciers and ice sheets. Low latitudes present stronger LAC strength and contribute more LAC area than the boreal middle and high latitudes. LAC hotspot regions in low latitudes are influenced by monsoons. LAC can be evident within the whole troposphere, while LAC is also detected in the lower stratosphere of low latitudes. In addition, the impact of LAC can even be detected in the upper stratosphere of mid-high-latitude. The seasonality of LAC strength is significant in all LAC regions. Globally, the boreal autumn and summer are the strong LAC period. The strongest LAC generally happens in autumn in the North Hemisphere, while in the boreal summer of the South Hemisphere. Significant ET flux is the key process in LAC, which transports the isotopic signal of soil moisture into atmospheric moisture and subsequently precipitation. In essence, moderate precipitation and energy input are requisite for strong LAC. At low latitudes, the four seasons are not clear with tropical and subtropical climates, and water and energy conditions are similar. Thus, there is no seesaw pattern of LAC strength between the two hemispheres. Compared to previous results, disagreements in hot spots and seasonality of LAC can be explained as the results of different spatiotemporal scales and environmental changes among studies.

Our LAC diagnosis method is a simple and efficient tool. Our study not only serves to further illustrate LAC but also serves as a starting point for the development of a universal isotopic tool for LAC diagnoses. In the future, our method could be applied to various spatiotemporal scales to verify the existence of an optimal scale. Besides, the strong LAC in high latitudes is highlighted in our result. However, relevant studies are currently rare. Given the sensitive responses to warming and significant feedback from LAC, attention is needed in future works.

### Supplementary Information

Supplementary Information.
