# Supplementary material for: Global diagnosis of land–atmosphere coupling based on water isotopes
Source: Sci Rep. 2023 Dec 3;13:21319. doi: 10.1038/s41598-023-48694-1 (PMC10694138; doi:10.1038/s41598-023-48694-1)
Supplement: Supplementary file 1 — Supplementary Information. [file 41598_2023_48694_MOESM1_ESM.docx]

Supporting Information for

**Global diagnosis of land-atmosphere coupling based on water isotopes**

Ruiqiang Yuan^1^*, Fei Li^1^, and Ruyu Ye

^1^School of Environment and Resource Sciences, Shanxi University, Taiyuan, China

^2^Institute of Geographic Sciences and Natural Resources Research, Chinese Academy of Sciences, Beijing, China

**Contents of this file**

Figures S1

Figures S2

Tables S1

**Introduction**

The annual air temperature, annual average humidity, and annual precipitation of the LAC hotspot regions are presented. The two groups are indicated in Figure S1.

The seasonality of LAC strength is significant. Globally, boreal autumn and summer are the period with strong LAC strength as shown in Figure S2.

Eleven LAC hotspot regions were recognized. Physical geographical conditions were calculated and summarized in Table S1 for every hotspot region of LAC.


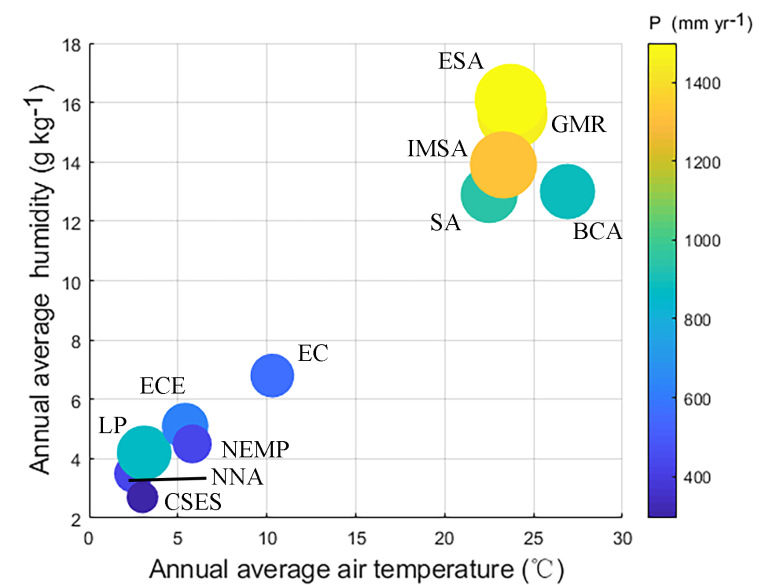


**Figure S1.** Grouping of the LAC hotspot regions


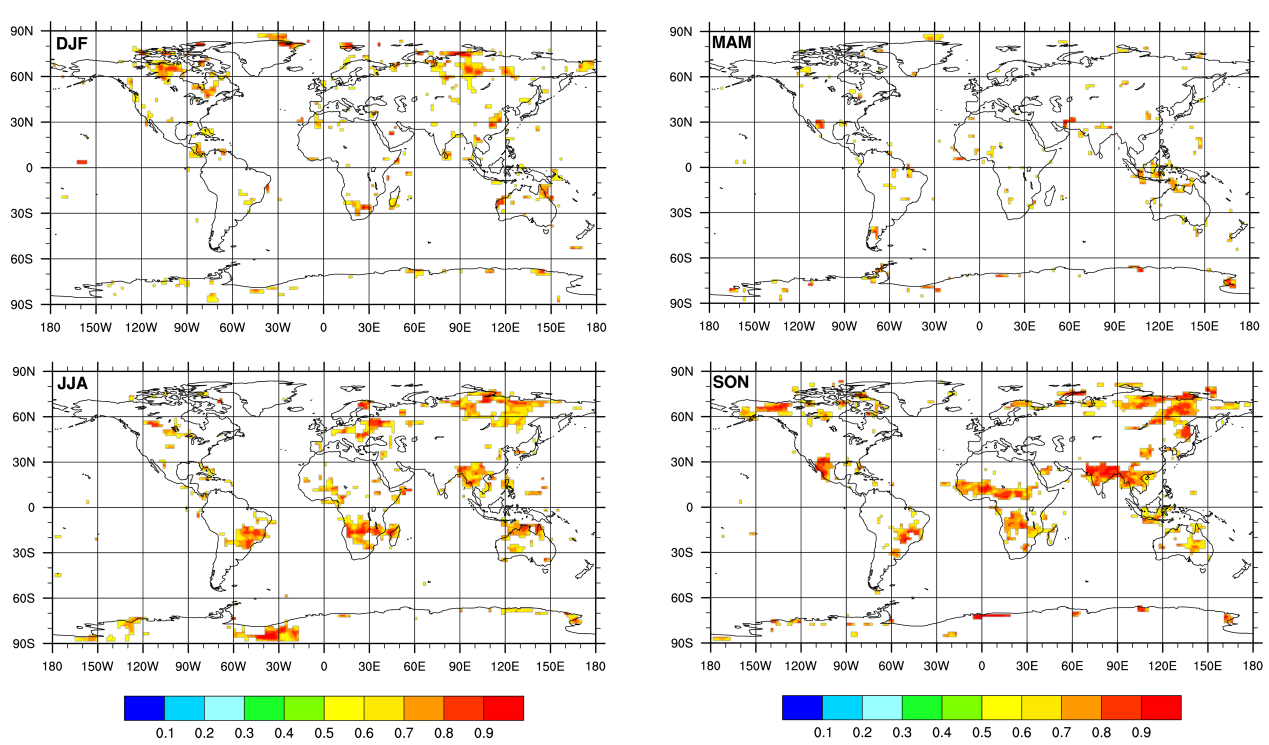


Figure S2. Seasonal variation of LAC strength

Table S1. Physical geographical conditions in the hotspot regions of LAC

| Regions | Area (r>0.5, 10^6^ km^2^) | Area (r>0.7, 10^6^ km^2^) | Averge precipitation (mm yr^-1^) | Annual average SMC^*^ (0-40cm, kg m^-2^) | Annual average humidity  (g kg^-1^) | Annual average air temperature (℃) | |
| --- | --- | --- | --- | --- | --- | --- | --- |
| NNA | 4.15 | 1.66 | 440 | 102.2 | 3.5 | 2.5 |  |
| LP | 1.23 | 0.19 | 873 | 121.4 | 4.2 | 3.1 |  |
| GMR | 2.94 | 1.45 | 1444 | 105.1 | 15.6 | 23.8 |  |
| ESA | 7.42 | 2.26 | 1499 | 108.9 | 16.1 | 23.7 |  |
| ECE | 3.95 | 0.99 | 632 | 116.8 | 5.1 | 5.4 |  |
| BCA | 7.32 | 3.15 | 895 | 90.9 | 13 | 26.9 |  |
| SA | 7.71 | 3.84 | 953 | 87.8 | 12.9 | 22.5 |  |
| CSES | 3.16 | 0.74 | 295 | 101.5 | 2.7 | 3 |  |
| NEMP | 4.83 | 2.65 | 444 | 91.5 | 4.5 | 5.8 |  |
| EC | 1.79 | 0.79 | 563 | 91.4 | 6.8 | 10.3 |  |
| IMSA | 7.83 | 5.03 | 1316 | 104.8 | 13.9 | 23.3 |  |

^*^ SMC, soil moisture content.

**Table S2. Long-term average precipitation for the LAC hotspots**

| No. | Regions | Long-term average precipitation in DJF (mm) | Long-term average precipitation in MAM (mm) | Long-term average precipitation in JJA (mm) | Long-term average precipitation in SON (mm) |
| --- | --- | --- | --- | --- | --- |
| 1 | NNA | 78 | 74 | 166 | 122 |
| 2 | LP | 165 | 170 | 277 | 261 |
| 3 | GMR | 154 | 255 | 564 | 471 |
| 4 | ESA | 594 | 454 | 137 | 314 |
| 5 | ECE | 134 | 124 | 204 | 170 |
| 6 | BCA | 26 | 168 | 444 | 257 |
| 7 | SA | 438 | 269 | 45 | 201 |
| 8 | CSES | 37 | 47 | 134 | 77 |
| 9 | NEMP | 28 | 75 | 246 | 95 |
| 10 | EC | 30 | 114 | 304 | 115 |
| 11 | IMSA | 50 | 193 | 726 | 348 |

**Table S3. Long-term average humidity for the LAC hotspots**

| No. | Regions | Long-term average humidity in DJF (g kg-1) | Long-term average humidity in MAM (g kg-1) | Long-term average humidity in JJA (g kg-1) | Long-term average humidity in SON (g kg-1) |
| --- | --- | --- | --- | --- | --- |
| 1 | NNA | 78 | 74 | 166 | 122 |
| 2 | LP | 165 | 170 | 277 | 261 |
| 3 | GMR | 154 | 255 | 564 | 471 |
| 4 | ESA | 594 | 454 | 137 | 314 |
| 5 | ECE | 134 | 124 | 204 | 170 |
| 6 | BCA | 26 | 168 | 444 | 257 |
| 7 | SA | 438 | 269 | 45 | 201 |
| 8 | CSES | 37 | 47 | 134 | 77 |
| 9 | NEMP | 28 | 75 | 246 | 95 |
| 10 | EC | 30 | 114 | 304 | 115 |
| 11 | IMSA | 50 | 193 | 726 | 348 |
